# How the Intricate Interaction among Toll-Like Receptors, Microbiota, and Intestinal Immunity Can Influence Gastrointestinal Pathology

**DOI:** 10.1155/2015/489821

**Published:** 2015-05-18

**Authors:** Simona Frosali, Danilo Pagliari, Giovanni Gambassi, Raffaele Landolfi, Franco Pandolfi, Rossella Cianci

**Affiliations:** Department of Medical Science, Institute of Internal Medicine, “A. Gemelli” University Hospital, Catholic University of the Sacred Heart, Largo A. Gemelli, 8-00168 Rome, Italy

## Abstract

The gut is able to maintain tolerance to microbial and food antigens. The intestine minimizes the number of harmful bacteria by shaping the microbiota through a symbiotic relationship. In healthy human intestine, a constant homeostasis is maintained by the perfect regulation of microbial load and the immune response generated against it. Failure of this balance may result in various pathological conditions. Innate immune sensors, such as Toll-like receptors (TLRs), may be considered an interface among intestinal epithelial barrier, microbiota, and immune system. TLRs pathway, activated by pathogens, is involved in the pathogenesis of several infectious and inflammatory diseases. The alteration of the homeostasis between physiologic and pathogenic bacteria of intestinal flora causes a condition called dysbiosis. The breakdown of homeostasis by dysbiosis may increase susceptibility to inflammatory bowel diseases. It is evident that environment, genetics, and host immunity form a highly interactive regulatory triad that controls TLR function. Imbalanced relationships within this triad may promote aberrant TLR signaling, critically contributing to acute and chronic intestinal inflammatory processes, such as in IBD, colitis, and colorectal cancer. The study of interactions between different components of the immune systems and intestinal microbiota will open new horizons in the knowledge of gut inflammation.

## 1. Introduction

The human gastrointestinal tract is colonized by different microbial populations including bacteria, fungi, and viruses [1]. Bacteria represent the largest population of intestinal microbiota, comprising 500–1000 different species [1, 2]. Surprisingly, the intestine is able to maintain tolerance to this antigenic burden, showing a symbiotic host relationship, and it may provide protective inflammatory responses against invading enteric pathogens. In fact, the intestinal tract has developed several strategies allowing a symbiotic relationship with microbiota and restricting the invasion of microorganisms through the gut epithelial barrier.

The intestine minimizes the number of harmful bacteria by shaping the microbiota through a symbiotic relationship. The commensal microbiota competes with pathogenic invaders and limits the colonization of the intestinal tract by pathogens [3, 4]. Furthermore, thick mucus layers composed of mucin glycoproteins secreted from Goblet cells create a physical barrier, which separates bacterial flora and the intestinal epithelial cells [5, 6]. Moreover, several antibacterial factors are secreted by mucosal cells and can directly regulate the microbiota's growth. For instance, Paneth cells at the base of the crypts are specialized cells that produce and secrete multiple antibacterial molecules, including *α*-defensins C-type lectins, lysozyme, and phospholipase A2 [7]. Secreted IgAs bind intestinal microorganisms, preventing their invasion through epithelial cell layers and controlling commensals. The IgA pools are antigen-specific [8] and are produced by plasma cells that migrate from Peyer's patches or other mucosal-associated lymphoid tissues, in response to epithelial signals.

In healthy human intestine, a constant homeostasis is maintained by the perfect regulation of microbial load and the immune response generated against it. Failure of this harmonized balance may result in various pathological conditions in the intestines. The alteration of the homeostasis between physiologic and pathogenic bacteria of intestinal flora causes a condition called dysbiosis. Thus, gut dysbiosis is a pathological condition characterized by an alteration of the normal bacterial flora that normally secretes vitamins, collaborates in digestion, regulates the permeability of the intestinal barrier, protects from infections, and prevents proliferation of pathogens. Consequently, the breakdown of homeostasis by dysbiosis or dysregulation of immune responses may increase susceptibility to Inflammatory Bowel Diseases (IBD) [9–11].

It is clear that innate immune sensors, such as Toll-like receptors (TLRs), play an important role in shaping intestinal microbiota. TLRs may be considered an interface among intestinal epithelial barrier, microbiota, and immune system. Moreover, TLRs pathway, activated by pathogens, is involved in the pathogenesis of several infectious and inflammatory diseases, such as IBD. In this review, we summarize the effects of TLRs on mucosal homeostasis and discuss the interaction between TLRs and human microbiota in the pathogenesis of gut diseases.

## 2. Toll-Like Receptors (TLRs)

TLRs are germline-encoded type I transmembrane receptors, expressed on numerous cell types including macrophages, dendritic cells (DCs), T lymphocytes, and intestinal epithelial cells. They act as pathogen recognition receptors (PRRs), identifying microbe-associated molecular pattern (MAMP), that are specific for microbes and essential for their survival [12]. TLR's name is derived from their similarity to the protein coded by the Toll gene identified in* Drosophila* in 1985. TLRs together with the Interleukin-1 receptors form a receptor superfamily, known as the “interleukin-1 receptor/Toll-like receptor superfamily” (Table 1).

A total of 10 TLRs are expressed in humans. Each TLR responds to distinct MAMPs, leading to the activation of specific signaling pathways. TLRs are characterized by the presence of an extracellular leucine-rich repeat domain (LRR) and an intracellular Toll/IL-1 receptor (TIR) domain [13]. LRRs are found on a diverse number of proteins and are involved in ligand recognition and signal transduction [14]. The TIR domain of the TLR is required for intracellular signaling and activation. This domain comprises about 200 amino acids, with varying degrees of sequence similarity among family members.

Three subgroups of TIR domains exist. Proteins of subgroup 1 are receptors for interleukins that are produced by macrophages, monocytes, and DCs; all these receptors have extracellular Immunoglobulin (Ig) domains. Proteins of subgroup 2 are considered a classical type of TLRs and bind directly or indirectly microbial molecules. Proteins of subgroup 3 are adaptor proteins, exclusively cytosolic, that mediate signals from proteins of subgroups 1 and 2 [14].

Activation of TLRs by their ligands induces several intracellular signaling cascades resulting in the production of cytokines and chemokines and in the transcription of other genes important for the control of infection. Two major signaling pathways have been detailed. The first pathway, which is the principal one, is activated by most TLRs and leads to activation of the transcription factor NF-*κ*B and the mitogen-activated protein (MAP) kinases p38 and JNK. These signaling cascades increase the expression of many proinflammatory genes. The second pathway is activated only by TLR3 and TLR4 and leads to activation of both NF-*κ*B and interferon regulatory factor 3 (IRF3) that is a transcription factor and induces an additional set of genes including antiviral genes, such as interferon-beta [14].

A central role in the TLR signaling is played by the adaptor molecules MyD88, MAL (also known as TIRAP), TRIF (also known as TICAM1), and TRAM (also known as TICAM2 or TIRP). MyD88 is used by all TLRs, except TLR3. MyD88 recruits IRAKs (IL-1R-associated kinase family), leading to the activation of MAP3 kinases. Two of MAP3 kinases have been identified, MEKK3 and TAK1; these activate NF-*κ*B, MAP kinases p38, and JNK. Activation of TAK1 by IRAKs requires TRAF6, as well as the ubiquitination of both TRAF6 and TAK1. BTK and PI3K also participate in TLR signaling.

Studies on MyD88-deficient mice have shown that signaling via TLRs plays an important role in intestinal homeostasis. This signaling is responsible for microbial recognition, induction of antimicrobial products, and modulation of the adaptive immune response [15, 16]. In fact, MyD88 knockout mice were susceptible to a greater number of bacterial infections caused by lack response of TLRs to MAMPs [17]. Moreover, recognition of commensal microbiota in a MyD88-dependent manner has been shown to be required for epithelial cell homeostasis [18], response to injury [19], and induction of antimicrobial peptides [20, 21].

## 3. TLR2 and Its Coreceptors (TLR1 and TLR6)

TLR2 is functionally expressed by distinct cell types in the intestinal mucosa and is constitutively expressed in the murine gastrointestinal epithelium, although this expression varies along the gut [22]. TLR2 recognizes a large spectrum of microbes, thanks to its ability to respond to molecular patterns such as lipoproteins [23, 24], lipoteichoic acid [25], and zymosan [26]. Ligand-induced activation of TLR2 leads to recruitment of TIRAP and MyD88, which results in activation of NF-*κ*B, and production of cytokines and chemokines [27, 28].

In healthy gut, the role of TLR2 in epithelial cells is to maintain tolerance to ubiquitous commensal lipoproteins. As a low TLR2 expression is important for tolerance, there are several mechanisms which contribute to maintain a low expression: (1) increased expression of negative regulators, such as Tollip and A20 [29]; (2) activation of cell signaling pathways [30] inducing production of anti-inflammatory IL-10. IL-10 inhibits macrophage and DC effector functions, limits immune responses [31], and promotes the local differentiation and activation of T-regulatory cells (Tregs).

In inflammatory disease, increase of TLR2 expression induces NF-*κ*B activation leading to exaggerated immune responses with production of inflammatory cytokines and such happens in Crohn's disease in which NOD2 is mutated [32].

Moreover, it is reported that TLR2 signaling conferred protection only against acute intestinal injury or inflammation [33], probably through maintenance of tight junction integrity [31]. In chronic inflammation, TLR2 showed moderate effects on regulation of sustained inflammatory processes [34, 35].

Furthermore, TLR2 signaling is critical for the acquisition of tissue-specific functional properties by gut-associated DCs, including their capacity to produce retinoic acid, to imprint gut-homing lymphocytes [36], and to activate Tregs. There is growing evidence about involvement of TLR2 in modulating T-cell functions both directly and indirectly. TLR2 stimulation can also promote T helper 17 cells (Th17) responses [37] and can reduce the suppressive function of Tregs by promoting a shift toward IL-17 production [38]. Notably, TLR2-induced mechanism of regulation of T-cell function could enhance microbial clearance and/or increase the risk of autoimmune reactions. However, commensal bacteria use a similar mechanism to enhance colonization of the gut and thereby establish host-microbial tolerance. For example,* B. fragilis* through TLR2, induces the production of the anti-inflammatory IL-10 in T-cells restraining Th17 responses [39]. Thus, TLR2, inducing pro- and anti-inflammatory effects, have a controversial action. The ability of TLR2 signaling to produce pro- and/or anti-inflammatory responses is influenced by the intestinal immunological niche, in which immune response, inflammation, and local homeostasis are modulated [40–42].

The complex response of TLR2 is further complicated by its ability to interact with multiple coreceptors [43], including TLR1 [44], TLR6 [44], Dectin-1 [45], CD36 [46], and CD14 [47].

For example, TLR6 associated with TLR2 uniquely induces IL-10 production by DCs and type-1 regulatory T-cells (Tr1). In contrast, TLR1 associated with TLR2 promotes differentiation of IL-12p40 production by DCs and inflammatory IFN-gamma T-cells (Th1) [48]. Furthermore, bacteria can also modulate the immune response based on the activation of TLR2 [49]. For example, bacterial triacylated lipoproteins activate TLR2/1, whereas bacterial diacylated lipoproteins activate TLR2/6, resulting in triggering different immune responses.

Thus, it is evident that the tissue microenvironment, bacteria composition, and metabolism all contribute to modulate the immune response.

## 4. TLR4

TLR4 is the best characterized pathogen-recognition receptor. Both immune cells and enterocytes express TLR4 [50]. Although there is a common signaling pathway and subsequent release of NF-*κ*B and IFN-beta [51], the downstream effects of TLR4 are varied. TLR4 is involved in both defense against pathogens and maintaining tolerance to commensal bacteria. Continuous recognition of selective commensals by TLR4 under steady-state conditions is essential in mucosal protection against exogenous injury [18].

Intestinal mucosa expresses low concentrations of TLR4 protein at baseline [52]. However, TLR4 expression is significantly increased in intestinal epithelial cells (IECs) and* lamina propria *mononuclear cells in association with acute inflammation, such as in IBD [22, 53, 54]. The presence of inflammatory cytokines such as IFN-gamma and TNF-alpha strongly upregulates TLR4 expression in IECs [55, 56]. During disruption of the epithelium, activation of TLR4 elicits inflammatory cytokine and chemokine expression with recruitment of innate and adaptive immune cells to limit bacterial invasion [57]. The absence of TLR4 signaling during injury results in a pattern of severe mucosal damage with impaired epithelial proliferation, attenuated inflammatory response, and marked bacterial translocation [58]. TLR4 signaling is important for induction of repair of the injured gut, so that increase in TLR4 expression may serve a protective role. However, in necrotizing enterocolitis, TLR4 activation has a strong role in the induction of mucosal injury in the newborn small intestine via increased enterocyte apoptosis and an inhibition in mucosal repair, through decreased enterocyte proliferation and migration [59, 60]. Moreover, Ungaro et al. have recently shown in a study using chimeric mice that TLR4 signaling in colonic epithelial cells worsened intestinal inflammation [61].

TLR4 signaling has been shown to affect the intestinal flora. Regulation of the microbiota by TLR4 appears to be attributable to alterations in gastrointestinal motility that drives clearance of pathogens and maintenance of commensal populations [62], differentiation of goblet cells [63], and expression of antimicrobial peptides. In mice, in response to alterations in the microbial flora of the gut, TLR4 may directly regulate transcription of defensin genes [64].

TLR4 can be affected by modification in diet. A high-fat diet induces dysregulation of the gut microbiota and activation of the TLR4 signaling pathway with consequent increased intestinal permeability [65].

Thus, the effects of TLR4 on intestinal mucosa are complicated. The appropriate or inappropriate TLR4 signaling is linked to a variety of factors, including the involved cells, cytokines and chemokines, and microenvironment.

## 5. TLR5

TLR5 is expressed on epithelial cells, endothelial cells, macrophages, DCs, and T-cells. TLR5 recognizes flagellin, the main protein of bacterial flagella, and is crucial for the detection of invasive flagellated bacteria at the mucosal surface [66]. TLR5 plays an important role in maintaining intestinal homeostasis by regulating host defense against enterobacterial infections. However, regulation of TLR5 expression and its function in the intestine have not been fully elucidated.

The work of Feng et al. has compared the expression of TLR5 in various human tissues. In particular, in intestinal mucosa, DCs express high levels of TLR5 with respect to splenic tissue. These differences are due to the different microenvironment of each tissue. In mucosa, host-derived factors such as retinoic acid and stromal cell products alter TLR5 expression [67]. Moreover, activation of TLR5 signaling induces differentiation of naive B-cells into plasma cells producing IgA and promotes development of antigen-specific Th1 and Th17 cells [67]. More recently, it has been reported that activation of TLR5 signaling induces mucosal production of IL-17 and IL-22; these interleukins promote early defenses against pathogen invasion of host tissues.

Furthermore, TLR5 signaling restricts Tregs generation but promotes effector T-cells. Therefore, high expression levels of TLR5 on* lamina propria* DCs give these cells a crucial role in the induction of effector T-cell responses against invading flagellated pathogens. On the other hand, DCs not expressing TLR5 may be responsible for maintenance of intestinal homeostasis, through induction of Tregs.

The recognition of flagellin by TLR5 is the principal mechanism through which the intestinal epithelia activate proinflammatory pathways in response to infections, such as* Salmonella enterica* [68]. However, studies on TLR5 knockout (TLR5KO) mice have shown that TLR5KO are resistant to* Salmonella* infection. This resistance has been attributed to changes in the basal phenotype of TLR5KO mice [68]. The small intestine and colon of these mice exhibit elevated levels of host defense genes that mediate innate and adaptive immunity in the gut. This includes changes in the basal phenotype of antimicrobial peptides and an increase in serum and fecal IgA and IgG and transport proteins in the gut [69]. TLR5KO mice have a homeostatic shift in microbiota composition with an increase in enterobacterial species, including* E. coli*, that was observed in proximity to the gut epithelium [70].

Thus, the absence of TLR5 signaling leads to increased resistance to infections and dysbiosis and leads to alterations in gene expression which then impact host metabolism. TLR5KO mice exhibit the hallmark features of a metabolic syndrome that includes hyperlipidemia, hypertension, insulin resistance, and increased adiposity. TLR5KO mice have insulin resistance even when on a calorie-restricted diet. It has been demonstrated that the transfer of TLR5KO microbiota to wild-type germ-free mice conferred many aspects of the TLR5KO phenotype, suggesting that the altered microbiota contributes to the development of the metabolic syndrome [71]. However, whether the altered microbiota is the cause or the effect in TLR5KO mice remains yet to be determined.

## 6. TLR9

TLR9 is expressed in antigen-presenting cells (APCs) including macrophages, DCs, and B lymphocytes. TLR9 is localized in the endosomal compartment and recognizes intracellular bacteria by binding unmethylated cytosine phosphate guanine (CpG) dinucleotides [72]. These nucleotides are expressed at high levels in prokaryotic DNA of commensal microbiota.

Activation of intracellular TLR9 drives the production of numerous proinflammatory cytokines, including TNF, IL-6, and IL-12, leading to a strong induction of the Th1-immune response [73]. Several studies have shown that TLR9 is effective in reducing apoptosis in gastrointestinal inflammatory disease. Studies examining the localization of TLR9 in intestinal epithelial cells have suggested that activation can occur via basolateral and apical surface domains of TLR9 [74]. These studies suggest that the signaling of TLR9 on the apical or basolateral surfaces determines whether the response is tolerogenic or inflammatory, respectively. Apical activation of TLR9 does not induce NF-*κ*B. In contrast, basolateral activation of TLR9 activates NF-*κ*B and ultimately induces IL-8 production [74].

The apical surface interacting with the intestinal lumen and coming into contact with commensal bacteria and probiotic DNA suppresses inflammation and it is protective in models of colitis. In fact, in models of experimental colitis, the administration of CpG significantly reduced the proinflammatory cytokine expression of IFN-gamma and IL-6, increased anti-inflammatory IL-10, and reduced disease severity [75]. In contrast to commensal bacteria, pathogenic bacteria that have breached the epithelium would stimulate basolateral TLR9 to produce inflammatory mediators and initiate the immune response. It was reported that TLR9 activation could limit TLR4 signaling in the gut, leading to reduced proinflammatory cytokines and apoptosis, thus ameliorating intestinal disease [76]. In the absence of TLR9, there is an increase in Tregs within the small intestine, leading to an inability to protect from infection [77].

## 7. TLRs Polymorphism in Human Gastrointestinal Pathology

Some data suggest that the human ability to respond to TLR ligands may be impaired by genetic variation within TLR genes, resulting in an altered susceptibility to infectious or inflammatory disease.

Genetic variations in TLRs may alter interaction between host and commensal bacteria. A defect in TLRs protein structure may influence ligand recognition, mucosal immune tolerance, and commensal composition, leading to innate/adaptive immune hypo- or hyperreactivity.

Several studies have evaluated the functional impact of TLR polymorphisms in IBD susceptibility. Although TLR polymorphism may not predict overall disease risk, they may influence phenotype severity in subgroups of patients with IBD [78].

The TLR variants are relatively rare. A number of variants in the* TLR1*,* TLR2, *and* TLR6* genes have been associated to distinct disease phenotypes of IBD. The polymorphisms TLR1-R80T and TLR2-R753Q in ulcerative colitis patients are associated with increased risk to develop pancolitis [79]. The SNPs TLR6-S249P was associated with a slightly decreased incidence of proctitis in IBD [79].

Allelic variants of the TLR4 gene may induce functional dysregulation of the lipopolysaccharide (LPS) receptor, exhibiting hyper- or hyposensitivity to LPS stimuli [80].

In active IBD, the allelic variants D299G and T399I exhibit proinflammatory effects in response to physiological concentrations of LPS [81, 82]. Increased susceptibility to IBD has been associated with the coexistence of TLR4 and/or NOD2 and BPI mutated alleles [11, 83]. An association between the TLR4-D299G polymorphism and sepsis has been also investigated. Two studies demonstrated that TLR4-D299G polymorphism increases the risk of gram-negative infections [84, 85] and another study linked this polymorphism to an increased incidence of systemic inflammatory response [86].

Several studies investigating associations between genetic variants of TLR genes and IBD have shown controversial results about TLR5 in human Crohn's disease.

Several mutations may induce an overrecognition of flagellin by TLR5 leading to intestinal inflammation. This could also explain the high prevalence of anti-flagellin antibodies in Crohn's disease patients compared to healthy controls. Recently, in one study, a partial functional dominant negative of TLR5 was associated with protection against Crohn's disease [87]; however the complete loss of TLR5(*TLR5* ^−/−^ mice) displays a high risk to develop colitis [88].

The gene encoding for TLR9 is mapped on chromosome 3p21.3 in the vicinity of a shared susceptibility locus for Crohn's disease and ulcerative colitis. Torok showed that genetic variation in TLR9 is associated with IBD [89]. The interactions between TLR9 polymorphisms and allelic variants in* NOD2* and* IL23R* differentially modulate susceptibility to Crohn's disease [90].

In addition, it should be noted that other genetic mutations and polymorphisms associated with genes and proteins involved in pathogenesis of gastrointestinal diseases such as NOD2, IL-10, MDR1-alpha, and STAT3 exist. They could interact with TLR polymorphisms, increasing the complexity of IBD.

## 8. The Role of TLRs and Its Interactions with Human Microbiota in the Pathogenesis of Inflammatory Bowel Diseases

IBD, comprising Crohn's disease and ulcerative colitis, are chronic and multifactorial diseases affecting the gastrointestinal tract. IBD are characterized by idiopathic intestinal inflammation, resulting from predisposing genetic (genes encoding proteins relevant to both innate and adaptive immunity: NOD2, STAT3, IL-23 receptor, etc.) and environmental factors (specific TLRs, ligands, and antigens derived from commensal bacteria) acting on the immunoregulatory system. IBD may be result of an imbalance of proinflammatory- and regulatory-T-cells responses [91]. The pathogenetic mechanism is still unknown. However, in genetically predisposed individuals there is an abnormal and inappropriate immune response against luminal agents (bacteria, viruses, and food), with the production of cytokines and other mediators of inflammation. Both humoral and cell-mediated immunity are involved in the pathogenesis of IBD. Then, cell-mediated immunity induces the activation of T-cells, macrophages, neutrophils, and other leukocytes.

Available evidence suggests that both dysregulated innate and adaptive immune pathways contribute to the aberrant intestinal inflammatory response in patients with IBD [92]. Most studies conducted in the last thirty years have focused on the role of abnormal adaptive immune responses in the pathogenesis of IBD. In particular, while Crohn's disease has long been considered to be driven by a Th1 response, ulcerative colitis has been rather associated with a nonconventional Th2 response [93]. Finally, it is important to consider that the innate immune response represents our first line of defense against pathogens [92].

It can be assumed that IBD are associated with an imbalance in the composition and function of intestinal bacterial flora. This involves, as a result of intestinal barrier dysfunction, a translocation of bacteria flora in the* lamina propria* and the activation of a strong inflammatory response following the activation of TLRs and of NF-*κ*B pathway, responsible for the transcription of various proinflammatory cytokines and chemokines [40, 92, 94]. This process is amplified by a decrease of the innate immune response that, in turn, determines a greater translocation of bacterial flora thorough the intestinal membrane. Overall, the progression of these diseases is due to a defect in immune regulation and immune tolerance in response to the initial inflammatory insult [94].

It has been noted that IBD probably have genetic components; they are not inherited in a Mendelian fashion and are thus probably due to a complex set of factors rather than solely to a gene. However, neither bacterial colonization nor genetics is sufficient to cause the disease, bacteria probably play a role in these disorders. Some suspect that IBD is due to a reduction in immune tolerance and subsequent overreaction of the host's immune system to harmful or nonharmful bacteria. Bacteria in the digestive tract may have pathogenic properties in addition to their health-inducing ones: they can produce toxins and carcinogens and have been implicated in such conditions as multisystem organ failure, sepsis, colon cancer, and IBD [2]. A major factor in health is the balance of bacterial numbers; if the numbers grow too high or low, it will result in harm to the host. The host has enzymes to regulate this balance. Some genera of bacteria, such as* Bacteroides* and* Clostridium*, have been associated with an increase in tumor growth rate, while other* genera*, such as* Lactobacillus* and* Bifidobacterium,* are known to prevent tumor formation [2].

On the other hand, some evidence demonstrated that bacteria help train the immune system; in addition, some forms of bacteria can prevent inflammation. Thus, the constant exposure of the intestinal mucosal surface to commensal derived TLR ligands induces a basal state of activation of downstream signaling pathways that ensures mucosal homeostasis through limited inflammatory responses and accelerated restitution and healing in the healthy intestine. Commensal composition and tolerance represent essential mechanisms of maintaining hyporesponsiveness of the intestinal immune system. The composition of the commensal microbiota depends on host immunity, genetics, and environment [78]. In return, the composition of the commensal microbiota actively shapes mucosal and systemic immune homeostasis of the host at multidimensional levels. The presence of commensals modulates TLR expression in the intestinal mucosa. The complexity of the commensal composition is critical in augmenting protective mucosal immunity [95]. Changes in the commensal composition may differentially modulate mucosal TLRs responsiveness, thus subverting immune responses to a predominantly proinflammatory phenotype. Both quantitative and qualitative changes in the microbial composition have been reported in IBD [96]. These bacterial changes in IBD patients contain abnormal compositions of the intestinal microbiota, characterized by reduced bacterial diversity, temporal instability, and depletion of distinct commensal species (members of the phyla Firmicutes and Bacteroidetes). The latter includes a lower proportion of* Faecalibacterium prausnitzii*, an anti-inflammatory commensal that counterbalances dysbiosis [78, 97]. Several causal scenarios are plausible in IBD pathogenesis but remain to be directly proven: genetic defects and/or aberrant immune-mediated modulation of specific TLRs may diminish antimicrobial activities and disturb bacterial clearance, leading to a colitogenic commensal composition. Changes in the commensal composition may subvert the mucosal innate immune system, leading to TLR-mediated hyper- or hyporeactive immune responses. Dysbiosis may inhibit effective TLR recognition and bactericidal activation [78].

Some bacteria have a pathogenic effect on gut homeostasis and infections may contribute to IBD pathogenesis. In fact, episodes of* Salmonella/Campylobacter* gastroenteritis have been associated with increased risk of developing IBD. Loss-of-function mutations in the TLR4 gene can predispose to these Gram-negative bacteria and increase susceptibility to enteric infection, which may represent an essential disease trigger in IBD pathogenesis. Pathogenic infections may change the commensal composition and disrupt commensal tolerance [98].* Campylobacter jejuni* may directly promote the internalization and translocation of commensal bacteria [99].

On the other hand, several negative control mechanisms that ensure tolerance to abundant resident microbiota and regulated activation via TLRs in the intestinal mucosa have recently been described: decreased surface receptor expression which limits frontline recognition, high expression levels of the downstream signaling suppressor Tollip, which inhibits IRAK activation, ligand-induced activation of PPARc (peroxisome proliferator-activated receptor c), which uncouples NF-*κ*B-dependent target genes in a negative feedback loop, negative regulation of proinflammatory IL-1R/TLR4 signaling through SIGIRR (single immunoglobulin IL-1R-related molecule; also known as TIR8), which abolishes exaggerated immune responses to commensal bacteria in colitis, ubiquitination of key TLR signaling components via ubiquitin-editing enzymes, such as A20, or E3 ubiquitin protein ligases, such as TRIAD3A, and selective induction of transcriptional repressors, such as Bcl-3, which limits proinflammatory responses via NF-*κ*B [78, 100]. Thus, inflammation in IBD may result from persistent commensal intolerance because of altered pattern recognition and TLR signaling. Accordingly, there is genetic evidence showing that the impaired recognition and killing of commensal bacteria also contribute to IBD development as has been suggested by the fact that many of the identified IBD-susceptibility genes regulate host-microbial interactions [94]. NOD2, which is an intracellular sensor of bacterial peptidoglycan, was identified as a susceptibility gene for Crohn's disease, and Crohn's disease-associated NOD2 mutations are associated with a loss of function of the protein [92, 94]. Three uncommon SNPs in NOD2 have been associated with susceptibility to ileal CD with an odds ratio equal to 2.4 in heterozygote individuals and 17.1 in homozygotes or compound heterozygotes, representing the strongest association with IBD to date [92]. Thus, defects in host mechanisms that recognize and clear bacteria are associated with the development of human IBD. How genetic defects lead to chronic colitis in patients with IBD remains unknown, but it is possible that impaired NOD2 or autophagy function might result in the accumulation of intestinal commensal bacteria that have the capacity to locally invade the intestinal mucosa and to trigger an abnormal inflammatory response [94].

Amongst the NOD family, in fact, NOD2 is crucially involved in IBD pathogenesis. It is expressed in the epithelium and senses muramyl dipeptide (MDP), which is a constituent of Gram-positive and Gram-negative bacteria [101]. Specific mutations of the NOD2 gene (Arg702Trp, Gly908Arg, and leu1007fsinsC) are linked to an increased susceptibility to ileal Crohn's disease [102]. The risk of developing ileal Crohn's disease is increased twofold to fourfold and 20-fold to 40-fold, respectively, for heterozygous and homozygous carriers of these NOD2 mutations [103]. In patients with NOD2 mutations, the activation of NF-*κ*B in response to MDP is defective, enabling bacteria to trigger inflammation [104]. TLRs detect microbiota and damage-associated molecular patterns and are involved in the maintenance of the commensal flora and mucosal homoeostasis. In the healthy intestine, TLRs are expressed in small amounts not only by epithelial cells, but also by monocytes, macrophages, and DCs [105].

IBD are also linked to good hygiene in youth, lack of breastfeeding, and consumption of large amounts of sucrose and animal fat [2]. In fact, in accordance with the fact that hygiene and the rate of infections in youth are connected to lifestyle and environment, the incidence and prevalence of IBD are high in industrialized countries with a high standard of living and low in less economically developed countries, having increased in developed countries throughout the twentieth century. IBD incidence is inversely linked to poor sanitation during the first years of life and consumption of fruits, vegetables, and unprocessed foods. Also, the use of antibiotics, which kill native gut flora and harmful infectious pathogens alike, especially during childhood, is associated with IBD.

Differential alteration of TLRs expression in IBD was first described at the beginning of the 21st century [78]. For example, TLR3 is downregulated in active Crohn's disease but not in ulcerative colitis and TLR5 is upregulated in both forms of IBD [78]. Essentially, these receptors provide a danger signal, which, amongst other effects, stimulates the formation of alpha- and beta-defensins [104, 106].

**Figure 1:**
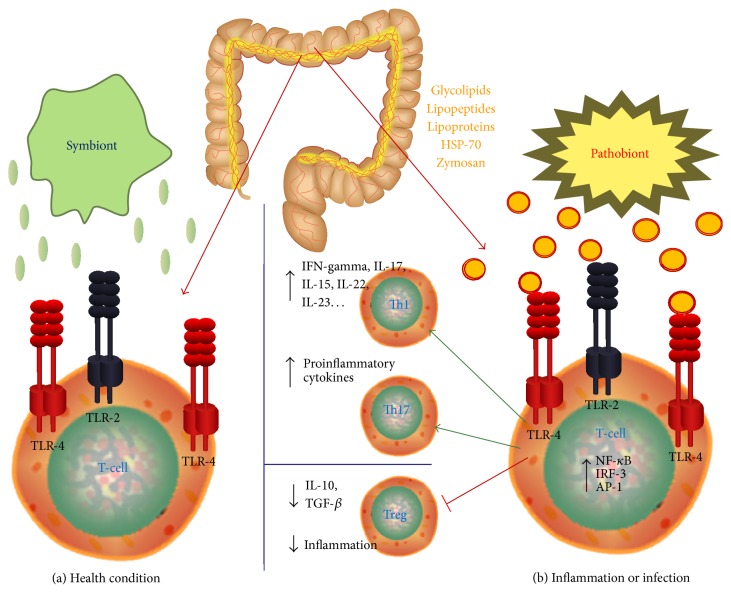
TLRs in human gastrointestinal pathology: health condition and inflammation. (a) In healthy human intestine, a constant homeostasis is maintained by the perfect regulation of microbial load and the immune response generated against it. (b) Failure of this balance may result in various pathological conditions. TLRs may be considered an interface among intestinal epithelial barrier, microbiota, and immune system. TLRs pathway, activated by pathogens, is involved in the pathogenesis of several infectious and inflammatory diseases. TLR signaling critically contributes to acute and chronic intestinal inflammatory processes.

In conclusion, the impact of TLR signaling on commensal-host interactions appears to be context-dependent. Environment, genetics, and host immunity modulate TLRs in the intestinal mucosa (Figure 1). Conversely, mucosal TLR signaling influences outcome of environmental signals, genetic functions, and immune responses in the intestine. There is an important dichotomy in TLRs regulation and function between healthy and inflamed intestinal mucosa, reflecting a fine line between host protection and destruction. In the healthy host, basal TLR signaling is significantly involved in protective host defense and tissue repair responses, crucially maintaining mucosal and commensal homeostasis. In the IBD-susceptible host, aberrant TLR signaling may contribute to destructive host responses and chronic inflammation, disturbing mucosal and commensal homeostasis and leading to many different clinical phenotypes. Hyperactivation of the adaptive immune system, secondary to TLRs deficiency, may drive tissue damage and progressive inflammation in IBD [78].

## 9. Conclusion

The small intestine has an enormous surface area that is continuously exposed to dietary and microbial antigens. These antigens need to be tolerated by the immune system to maintain homeostasis. This important role is played by immune sensors such as TLRs. Unfortunately, in some cases the innate immune system fails to protect the host, and chronic inflammation and other disorders occur.

It is evident that environment, genetics, and host immunity form a multidimensional and highly interactive regulatory triad that controls TLR function in the intestinal mucosa. Imbalanced relationships within this triad may promote aberrant TLR signaling, critically contributing to acute and chronic intestinal inflammatory processes, such as in IBD, colitis, and colorectal cancer.

Changes in intestinal microbiota through genetics and environment may contribute to defective host immune response.

The gut microbiota has been studied for a long time. Recent studies have shown ever-expanding roles for these microscopic organisms in health and disease. Despite the complexity of microbial population present in gut, a delicate balance between host and bacteria populations exists. The disruption of this balance leads to dysbiosis and, consequently, to decreased resistance to pathogen colonization, to the favored growth of pathobionts, and to pathological immune responses by the host.

In many diseases, including IBD, dysbiosis is an important immunologic pathogenenic process. Dysbiosis and immune dysregulation might have a greater influence in young children than adolescents or adults. However, it is not clear whether dysbiosis contributes to the development of IBD or is instead a consequence of the disease. Indeed, antibiotics are not effective in the treatment of IBD, except in specific circumstances. For this reason, a better knowledge of the mechanisms underlying the intestinal innate immune response is crucial for developing of new therapies and vaccines to protect against pathogens and chronic inflammation.

Moreover, the understanding of host-microbial immune mutualism is fundamental because it is intimately connected with human health. Thus, the study of interactions between different components of the innate and adaptive immune systems, especially in relationship with the intestinal microbiota, will open new horizons in the knowledge of gut inflammation mechanisms.

## Figures and Tables

**Table 1:** Human TLRs: an overview on their pathophysiology.

	Ligand(s)	Cell types	Genetic defect	Association
TLR 1	(i) Triacyl lipopeptides	(i) Monocytes/macrophages (ii) Dendritic cells (iii) B lymphocytes	TLR1-R80T	Ulcerative colitis, pancolitis

TLR 2	(i) Glycolipids (ii) Lipopeptides (iii) Lipoproteins (iv) Lipoteichoic acid (v) HSP-70 (vi) Zymosan (vii) Others	(i) Monocytes/macrophages (ii) Neutrophils (iii) Myeloid dendritic cells (iv) Mast cells	TLR2-R753Q	Ulcerative colitis, pancolitis

TLR 6	(i) Diacyl lipopeptides	(i) Monocytes/macrophages (ii) Mast cells (iii) B lymphocytes	TLR6-S249P	Decreased incidence of proctitis in IBD

TLR 4	(i) Lipopolysaccharide (ii) Heat shock proteins (iii) Fibrinogen (iv) Heparan sulfate (v) Hyaluronic acid (vi) Nickel (vii) Various opioid drugs	(i) Monocytes/macrophages (ii) Neutrophils (iii) Myeloid dendritic cells (iv) Mast cells (v) B lymphocytes (vi) Intestinal epithelium	TLR4-D299G	Increased susceptibility to IBD

TLR 5	(i) Flagellin	(i) Monocyte/macrophages (ii) Dendritic cells (iii) Intestinal epithelium	TLR5-STOP	Decreased susceptibility to IBD

TLR 9	(i) Unmethylated CpG (ii) Oligodeoxynucleotide DNA	(i) Monocytes/macrophages (ii) Plasmacytoid dendritic cells (iii) B lymphocytes	TLR9-SNPs: −1237T/C −2848A/G	Susceptibility to Crohn's disease
